# Extreme temperatures and sickness absence in the Mediterranean province of Barcelona: An occupational health issue

**DOI:** 10.3389/fpubh.2023.1129027

**Published:** 2023-02-20

**Authors:** Mireia Utzet, Amaya Ayala-Garcia, Fernando G. Benavides, Xavier Basagaña

**Affiliations:** ^1^Center for Research in Occupational Health (CiSAL), Universitat Pompeu Fabra, Barcelona, Spain; ^2^Hospital del Mar Medical Research Institute (IMIM), Barcelona, Spain; ^3^Center for Biomedical Research Network (CIBER) of Epidemiology and Public Health, Madrid, Spain; ^4^Institute for Global Health (ISGlobal), Barcelona, Spain; ^5^Departament de Medicina i Ciències de la Vida, Universitat Pompeu Fabra, Barcelona, Spain

**Keywords:** temperature, sickness absence, cold, heat, prevention, Mediterranean

## Abstract

**Objectives:**

This study aims to assess the association between daily temperature and sickness absence episodes in the Mediterranean province of Barcelona between 2012 and 2015, according to sociodemographic and occupational characteristics.

**Methods:**

Ecological study of a sample of salaried workers affiliated to the Spanish social security, resident in Barcelona province between 2012 and 2015. The association between daily mean temperature and risk of new sickness absence episodes was estimated with distributed lag non-linear models. The lag effect up to 1 week was considered. Analyses were repeated separately by sex, age groups, occupational category, economic sector and medical diagnosis groups of sickness absence.

**Results:**

The study included 42,744 salaried workers and 97,166 episodes of sickness absence. The risk of sickness absence increased significantly between 2 and 6 days after the cold day. For hot days there was no association with risk of sickness absence. Women, young, non-manual and workers in the service sector had a higher risk of sickness absence on cold days. The effect of cold on sickness absence was significant for respiratory system diseases (RR: 2.16; 95%CI: 1.68–2.79) and infectious diseases (RR: 1.31; 95%CI: 1.04–1.66).

**Conclusion:**

Low temperatures increase the risk of having a new episode of sickness absence, especially due to respiratory and infectious diseases. Vulnerable groups were identified. These results suggest the importance of working in indoor and possibly poorly ventilated spaces in the spread of diseases that eventually lead to an episode of sickness absence. It is necessary to develop specific prevention plans for cold situations.

## Introduction

The Intergovernmental Panel on Climate Change has reported evidence on the environmental consequences of climate crisis, such as global warming, sea-level rise, heat waves, heavier hurricanes and storms, and has projected a global average temperaturet increase of 1.8 to 4°C by 2100 (1). Climate crisis has different effects in different areas of the world. For instance, the Mediterranean region has been identified as a critical region for the climate change (2), and will probably experience an intensification of summer maximum temperature warming, although a less pronounced warming of winter minimum temperature (3).

Extreme temperatures, which will be aggravated by global warming, have been identified as a major public health concern, with significant impacts on human health (4). The multiple health impacts of extreme high and low temperatures have been well established, including an increase in infectious, cardiovascular and respiratory diseases and general mortality (5). Furthermore, some groups are especially vulnerable to climate crisis health effects, such as women, children and the elderly (6), and people with chronic diseases (7). Most of the existing studies on the effects of extreme temperature have focused on mortality and hospital admissions, which represent the most serious health consequences. These effects of ambient temperature are mainly concentrated in the elderly. However, ambient temperatures could also be linked to other, milder health conditions that can also be suffered by a younger population.

In the occupational setting, extreme temperatures have been linked with other health effects metrics. For example, the relationship between heat exposure and some illnesses (e.g., kidney disease and acute kidney injury) have been increasingly studied in the working population (8–10) and the evidence from various countries indicates that extreme heat may contribute to an increased risk of occupational morbidity, mortality and injuries (11, 12). Moreover, some studies indicate some effects of cold exposure on the risk of occupational injury (12, 13).

Sickness absences can be a good metric to evaluate the health effects of temperature in the working population. In a seminal work, Marmot and colleagues have considered sickness absence as an integrated measure of health status and functioning in working population (14). Poor working conditions may contribute to sickness absence, acting both as the cause of an occupational disease or accident at work or interacting with a common disease, leading to a sickness absence (15). The relationship between extreme temperatures and sickness absence has rarely been studied, but it has been shown that SA is influenced by season, being sickness absence rates higher in winter than in summer (16, 17). As suggested by Pocock, the impact of temperature on sickness absence incidence could be understood as result of a direct physiological effect, deteriorating individuals' health, or of a social effect, whereby a change in social behavior indirectly increases the spread of an infection (16). Others employment and working conditions, and also individual characteristics could aggravate or attenuate this impact.

Therefore, the aim of this study is to assess the possible association between daily temperature and incidence of non-work-related sickness absence in Barcelona province between 2012 and 2015, according to sociodemographic and occupational characteristics.

## Methods

### Setting and design

We performed an ecological study of a sample of salaried workers residents in the province of Barcelona, who are affiliated at least 1 day to the general regime of the Spanish social security between 2012 and 2015, and have had at least one episode of sickness absence. The province of Barcelona has a population of approximately 5.5 million. The predominant climate in the region is Mediterranean, with dry, hot summers and winters with balanced temperatures and low rainfall.

Data sources were the WORKss cohort (18), which is part of the Continuous Working Life Sample (CWLS), an annual random representative sample of 4% of affiliates of the Spanish social security system for the whole Spain, and the Catalan Institute of Medical and Health Evaluations, which registers all sickness absence episodes among social security affiliates in Catalonia and provided SA records. Furthermore, temperature daily data were obtained from the Catalan Meteorological Institute (METEOCAT) (19). In particular, from the “el Raval” official meteorological station, a neighborhood located in the city of Barcelona.

### Study variables

The main outcome is sickness absence, a situation in which a worker affiliated to the Social Security is unable to carry out his or her work, no more than one and a half year, meanwhile is receiving health care from a health care center and an economic subsidize from the social security system (20). We aggregated new sickness absence by date, and daily counts were also calculated separately by sex (women, men), age (<25, 26–35, 36–45, 46–55, and more than 55 years), economic sectors (primary: agriculture, hunting, forestry, fishing, mining, and quarrying; secondary: manufacturing, energy construction; and tertiary: all services), medical diagnosis of sickness absence based on the ICD-10 revision (Infectious diseases A00–B99; Mental disorders F00–F99; Respiratory system diseases J00–J99; Digestive system diseases K00–K93; Musculoskeletal system and connective tissue diseases M00–M99; Diseases of the genitourinary system N00–N99; Signs, symptoms and abnormal findings R00–R99; Trauma, poisoning and other consequences of external causes S00–T98; and Other -every other ICD-10 revision that doesn't fall into the ones mentioned before), and occupational category (manual and non-manual). Weather data was assessed by the daily minimum, average and maximum temperature.

### Statistical analysis

Daily new sickness absence episode counts were linked with temperature using quasi-Poisson regression and the distributed lag non-linear model (dlnm) framework (21). In particular, we used the penalized framework for distributed lag non-linear models (22), which facilitates the choice of smoothing parameters. The possible exposure-response association was modeled using a P-spline with 9 degrees of freedom. The possible lag-response association was modeled using a P-spline with 10 degrees of freedom and a maximum lag of 7. We presented the cumulative (over all lags) exposure-response functions and relative risks (RR) for cold (taking the 5th percentile of temperature as representative) and hot (taking the 95th percentile of temperature) temperatures taking as reference (centering point) the temperature of minimum risk. Additional analyses were performed, taking the 1st and the 99th percentiles of temperature as representatives, and results remained largely the same (data not shown).

We controlled for long-term trends and seasonality by including a natural spline of time using 12 degrees of freedom per year. This choice was based on visually inspecting how the splines with different degrees of freedom captured the seasonal pattern in the data. We also compared the estimated seasonal pattern with different degrees of freedom with that obtained using a thin plate regression spline of time. Since the daily number of sickness absence depends on the number of workers working on a given day, we also included dummy variables for day of the week holidays, and certain days preceding or following a holiday. The inclusion of such days was decided by aggregating all sickness absence episodes by day of year and visually exploring the resulting time series plot. Those points that appeared separated from the seasonal pattern, and were a holiday or a day preceding or following a holiday, were included as dummy variables in the model.

Finally, we calculated the attributable fraction of sickness absence that could be attributed to non-optimal temperatures based on the model results using a previously established methodology (21). The results were stratified by sex, age, occupational category, economic sectors and medical diagnosis groups. Additional analyses were repeated separately by women and men, but results were similar (data not shown).

### Ethics

This study was performed in accordance with the standards of Good Clinical Practice and the principles of the Declaration of Helsinki. The study protocol guaranteed the fulfillment of Regulation (EU) 2016/679 of the European Parliament and the Council of 27 April 2016 on the protection of natural persons regarding the processing of personal data and the free movement of such data.

## Results

The study included 42,744 salaried workers and 97,166 episodes of sickness absence registered in Barcelona province between 2012 and 2015 (Table 1). These episodes occurred at an average of 66.51 (Standard Deviation: 52.2) sickness absence episodes per day. Overall, women, individuals aged 26–35, and non-manual workers registered more sickness absence episodes (Table 1), and these were more frequent in winter. The daily average minimum temperature during the study period was 15.2°C (Standard Deviation: 5.8) and it ranged from 0.6°C to 26.8°C. The daily average maximum temperature was 21.4°C (Standard Deviation: 5.6), ranging from 5.6°C to 35.6°C. These results reflect the mild temperatures in Barcelona province.

**Table 1:** Distribution of a sample of salaried workers residents in Barcelona who had a sickness absence (SA) episode in Barcelona between 2012 and 2015 by sex, age, occupational category, economic sectors, and medical diagnosis.

		**Total**		**2012**		**2013**		**2014**		**2015**	
		***n*** **(%)**	**SA episodes (%)**	***n*** **(%)**	**SA episodes (%)**	***n*** **(%)**	**SA episodes (%)**	***n*** **(%)**	**SA episodes (%)**	***n*** **(%)**	**SA episodes (%)**
Sex	Men	19,365 (45.3)	40,788 (42.0)	4,967 (45.3)	10,167 (42.2)	4,452 (45.2)	9,465 (41.5)	4,605 (45.8)	9,966 (42.2)	5,341 (45.0)	11,190 (42.0)
	Women	23,379 (54.7)	56,378 (58.0)	5,989 (54.7)	13,929 (57.8)	5,399 (54.8)	13,325 (58.5)	5,452 (54.2)	13,667 (57.8)	6,539 (55.0)	15,457 (58.0)
Age	<25	5,191 (12.1)	11,660 (12.0)	923 (8.4)	2,166 (9.0)	952 (9.7)	2,426 (10.6)	1,286 (12.8)	2,927 (12.4)	2,030 (17.1)	4,141 (15.5)
	26–35	13,039 (30.5)	31,174 (32.1)	3,277 (29.9)	7,601 (31.5)	2,980 (30.3)	7,253 (31.8)	3,129 (31.1)	7,721 (32.7)	3,653 (30.8)	8,599 (32.3)
	36–45	12,357 (28.9)	27,796 (28.6)	2,224 (29.4)	7,085 (29.4)	2,952 (30.0)	6,591 (28.9)	2,875 (28.6)	6,726 (28.5)	3,306 (27.8)	7,394 (27.7)
	46–55	8,418 (19.7)	18,616 (19.2)	2,207 (20.1)	4,706 (19.5)	2,023 (20.5)	4,463 (19.6)	2,014 (20.0)	4,549 (19.2)	2,174 (18.3)	4,898 (18.4)
	>55	3,739 (8.8)	7,92 (8.2)	1,325 (12.1)	2,538 (10.5)	944 (9.6)	2,057 (9.0)	753 (7.5)	1,71 (7.2)	717 (6.0)	1,615 (6.1)
Occupational category	Manual	16,792 (39.5)	36,721 (37.9)	4,254 (39.2)	8,995 (37.5)	3,780 (38.7)	8,496 (37.5)	3,960 (39.5)	8,953 (38.0)	4,798 (40.5)	10,277 (38.6)
	Non-manual	25,723 (60.5)	60,133 (62.1)	6,609 (60.8)	14,991 (62.5)	5,996 (61.3)	14,192 (62.5)	6,057 (60.5)	14,619 (62.0)	7,061 (59.5)	16,331 (61.4)
Economic sector	Agriculture, hunting, forestry, fishing, mining, and quarrying	177 (0.4)	332 (0.3)	45 (0.4)	87 (0.4)	41 (0.4)	72 (0.3)	38 (0.4)	72 (0.3)	53 (0.5)	101 (0.4)
	Manufacturing, energy construction	8,198 (19.2)	17,952 (18.5)	2,136 (19.5)	4,493 (18.7)	1,978 (20.1)	4,299 (18.9)	1,899 (18.9)	4,307 (18.2)	2,185 (18.4)	4,853 (18.2)
	Services	34,140 (79.9)	78,570 (80.9)	8,682 (79.2)	19,406 (80.5)	7,757 (78.7)	18,317 (80.4)	8,080 (80.3)	19,193 (81.2)	9,621 (81.0)	21,654 (81.3)
Medical diagnosis groups (ICD-10)	Infectious diseases (A00–B99)	4,841 (11.3)	12,558 (12.9)	1,356 (12.4)	3,351 (14.0)	1,008 (10.2)	2,811 (12.3)	1,062 (10.6)	2,851 (12.1)	1,415 (11.9)	3,545 (13.3)
	Mental disorders (F00–F99)	2,824 (6.6)	6,530 (6,7)	730 (6.7)	1,556 (6.5)	689 (7.0)	1,563 (6.9)	646 (6.4)	1,612 (6.8)	759 (6.4)	1,799 (6.8)
	Respiratory system diseases (J00–J99)	8,872 (20.8)	20,839 (21.5)	2,079 (19.1)	4,894 (20.4)	2,208 (22.4)	5,044 (22.1)	2,006 (20.0)	4,993 (21.1)	2,579 (21.7)	5,908 (22.2)
	Digestive system diseases (K00–K93)	2,661 (6.2)	6,124 (6.3)	715 (6.6)	1,536 (6.4)	568 (5.8)	1,400 (6.1)	602 (6.0)	1,440 (6.1)	776 (6.5)	1,748 (6.6)
	Musculoskeletal system and connective tissue diseases (M00–M99)	8,347 (19.6)	18,414 (19.0)	2,083 (19.1)	4,411 (18.4)	1,888 (19.2)	4,274 (18.8)	2,054 (20.4)	4,625 (19.6)	2,322 (19.6)	5,104 (19.2)
	Signs, symptoms and abnormal findings (R00–R99)	3,295 (7.7)	8,365 (8.6)	857 (7.9)	2,138 (8.9)	744 (7.6)	1,885 (8.3)	773 (7.7)	2,056 (8.7)	921 (7.8)	2,286 (8.6)
	Trauma, poisoning and other consequences of external causes (S00–T98)	4,150 (9.7)	7,706 (7.9)	1,023 (9.4)	1,823 (7.6)	939 (9.5)	1,811 (8.0)	1,039 (10.3)	1,984 (8.4)	1,149 (9.7)	2,088 (7.8)
	Diseases of the genitourinary system (N00–N99)	1,590 (3.7)	3,529 (3.6)	354 (3.3)	792 (3.3)	367 (3.7)	847 (3.7)	404 (4.0)	888 (3.8)	465 (3.9)	1,002 (3.8)
	Other diagnoses	6,108 (14.3)	12,968 (13.4)	1,708 (15.7)	3,468 (14.5)	1,435 (14.6)	3,149 (13.8)	1,471 (14.6)	3,184 (13.5)	1,494 (12.6)	3,167 (11.9)

**Figure 1:**
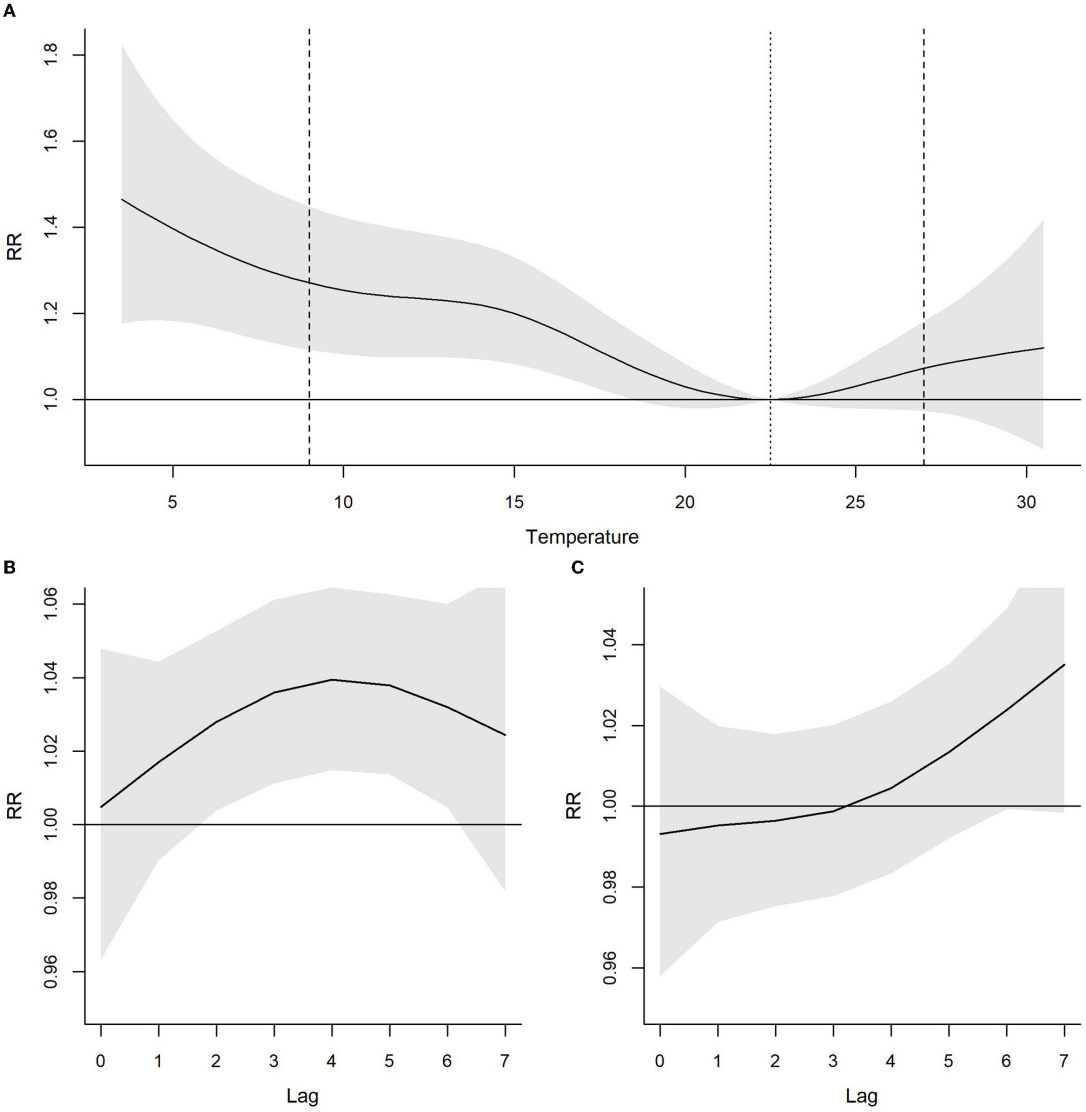
Relationship between average temperature (°C) and risk of sickness absence in Barcelona for the period 2012–2015 with 95% confidence intervals. **(A)** Cumulative Relative Risks (RR) for lags 0–7. The dashed lines indicate the 5th and 95th percentiles of average temperature. The dotted line indicates the temperature of minimum risk. **(B)** Association of cold temperatures (RR for 5th percentile of temperature compared to temperature of minimum risk) by lag. **(C)** Association of hot temperatures (RR for 95th percentile of temperature compared to temperature of minimum mortality) by lag.

The relationship between temperature and risk of sickness absence is summarized by the pooled overall cumulative curve shown in Figure 1A. The curve indicates that the risk of sickness absence significantly increases in cold temperatures (0°C−17°C approximately), but not at high temperatures. Taking the 5th percentile temperature of the whole period as representative of a cold day (i.e., 10°C), and 21°C as the reference category, the sickness absence risk increased during the period of 2 to 6 days after the cold day (lag 2–6, Figure 1B); in the case of hot days, there was no association with the sickness absence risk (Figure 1C). Cumulating the effect of all the lags, cold days increased the risk of sickness absence by 25% (95% CI 11–42).

When stratifying by the sociodemographic and occupational variables, still we did not find statistically significant associations for heat (Table 2). The association between cold temperatures and the overall sickness absence risk presented differences in terms of sex, occupational category and economic activity. In particular, women, workers younger than 45 years old, non-manual and workers in the services sector presented significant RRs. Lastly, when stratifying by medical diagnosis of sick leave, the association between cold temperatures and the risk of sickness absence was only statistically significant for respiratory system diseases (RR: 2.16, 95% CI: 1.68–2.79) (Supplementary Figure S1) and infectious diseases (RR: 1.31, 95% CI: 1.04–1.66) (Supplementary Figure S2). For respiratory system diseases, the cold effect was observed beyond 7 days, in particular it was observed after 11 to 12 days after the cold day (data not shown).

**Table 2:** Relative Risk (95% confidence intervals) for the association between cold and heat and sickness absence in Barcelona by sex, age, occupational category, economic activity and cause of sickness absence for the period 2012–2015.

		**Cold**	**Heat**
Total		1.25 (1.11–1.42)	1.07 (0.97–1.18)
Sex	Men	1.16 (0.99–1.35)	1.09 (0.96–1.23)
	Women	1.32 (1.14–1.53)	1.04 (0.94–1.15)
Age	<45	1.25 (1.1–1.43)	1.08 (0.98–1.20)
	>45	1.22 (0.98–1.52)	1.00 (0.97–1.03)
Occupational category	Manual	1.15 (0.92–1.44)	1.02 (0.99–1.06)
	Non-manual	1.31 (1.14–1.51)	1.07 (0.96–1.19)
Economic activity	Agriculture, hunting, forestry, fishing, mining, and quarrying	-	-
	Manufacturing, energy, construction	1.03 (0.88–1.22)	0.98 (0.90–1.07)
	Services	1.29 (1.13–1.47)	1.05 (0.95–1.15)
Cause of sickness absence	Mental health disorders	1.19 (0.89–1.59)	1.05 (0.82–1.35)
	External diseases	1.02 (0.81–1.29)	0.99 (0.87–1.13)
	Digestive system diseases	0.94 (0.71–1.23)	1.06 (0.86–1.32)
	Musculoskeletal system and connective tissue diseases	1.05 (0.90–1.24)	0.95 (0.86–1.06)
	Diseases of the genitourinary system	0.98 (0.64–1.49)	1.12 (0.77–1.62)
	Signs, symptoms and abnormal findings	1.10 (0.89–1.37)	0.95 (0.84–1.07)
	Trauma, poisonning and other consequences of external causes	1.02 (0.81–1.29)	0.99 (0.87–1.13)
	Respiratory diseases	2.16 (1.68–2.79)	1.09 (0.86–1.37)
	Infectious diseases	1.31 (1.04–1.66)	1.25 (0.97–1.60)
	Other diagnoses	0.92 (0.75–1.12)	1.06 (0.90–1.24)

Table 3 shows the number and percentage of sickness absence episodes attributable to infectious and respiratory diseases attributable to overall non-optimal temperatures, cold and heat. More than 11,500, that represent the 55.3% (41.5–64.7) of the sickness absence episodes registered in the 4-year study period due to respiratory diseases, were attributable to non-optimal temperatures, mainly to cold temperatures. Around 2,100 [17.1% (7.7–25.8)] of the sickness absence episodes due to infectious diseases were attributable to non-optimal temperatures, mostly cold.

**Table 3:** Measures of attributable fraction of sickness absence episodes and days of leave (95% Confidence Interval) due to respiratory and infectious diseases, computed as overall non-optimal temperatures and separately for cold and heat in Barcelona.

		**Total**	**Cold**	**Heat**
Respiratory diseases	Attributable no sickness absence	11,517 (8,641–13,473)	11,401 (8,289–13,436)	116 (−69–258)
	Attributable risk (95%CI)	55.3 (41.5–64.7)	54.7 (39.8–64.5)	0.6 (−0.3–1.2)
Infectious diseases	Attributable no sickness absence	2,142 (962–3,243)	1,827 (484–2,886)	315 (−71–645)
	Attributable risk (95%CI)	17.1 (7.7–25.8)	14.6 (3.9–23.0)	2.5 (−0.6–5.1)

## Discussion

This article aimed to assess the association between daily temperature and sickness absence episodes in Barcelona province. Our results show that low temperatures increase the risk of having an episode of sickness absence, especially due to respiratory and infectious diseases. Furthermore, women, non-manual workers and workers in the service sector seemed to be more susceptible to cold temperatures. No association was found between hot temperatures and risk of sickness absence.

Climate crisis indicators, such as meteorological variables, have been considered as macro determinants of sickness absence (23), and have hardly been analyzed. However, sickness absence is a complex phenomenon that affects quality of life and economics at different structural levels. Both the theoretical development and the amount of evidence-based knowledge regarding the occurrence and etiology of sick have grown over the last decades, but are still modest (24). Improving knowledge of the determinants of sickness absence is crucial to target effective measures to improve the quality of life of workers and reduce costs for employers and social security (25).

The main result is that cold temperatures increased the risk of sickness absence. This result is coherent with other publications which showed that cold exposure increased the risk of occupational injury (12, 13). Moreover, although little research has been carried out on the effect of cold waves on mortality, it has been shown that, in Spain, cold-related morbidity and mortality in the general population can be higher than heat-related (26). As we have analyzed a younger and healthier population, our results suggest that cold has more effects than usually reported, and in a larger population.

Unexpectedly, we did not find association between hot temperatures and risk of sickness absence. Most of previous studies analyzing temperature effects in occupational health showed that heat and extremely heat temperatures increased the risk of occupational injuries (5, 10, 12). This unexpected result can be partly explained because we are not accounting for health problems derived from an occupational injury that are mostly recognized as a work-related sickness absence. Furthermore, it may be related to the characteristics of the cohort, this is, mainly workers at services sector who are presumably in regulated indoor climates during their working time, and almost no agriculture or construction workers, who have high rates of traumatic injury and high risks of adverse heat health outcomes (10) due to the precarious working and living conditions they are exposed to. So, further research is needed to understand this unexpected pattern. Mainly, at this current time when high temperatures are dramatically increasing in the South of Europe and the Mediterranean basin (27).

We found an association between cold temperatures and sickness absence due to respiratory and infectious diseases, both groups of diseases strongly interrelated by infectious diseases of the upper respiratory tract. This result is in line with other studies on temperature and hospitalizations or occupational injuries (28). Bronchitis, influenza, and upper respiratory illnesses, common during winter, are the main causes for sickness absence from December to March (29). However, our analyses removed seasonal trends and therefore it captured associations that remain after those trends (e.g., influenza season). Moreover, gastroenteritis caused by norovirus has been pointed out as the cause of most of sickness absence episodes due to infectious diagnoses (30). Both associations may be facilitated both by cold and humid air (31) and by more hours spent indoors without proper ventilation, with a closer proximity that increases the probability of worker-to-worker transmission of airborne pathogens (32). Thus, the association may be partly mediated by the circulation of pathogens and not caused by the direct effect of cold temperatures on workers' health.

We identified especially vulnerable groups in the association of daily temperature and the risk of having a sickness absence episode. Women, regardless of age, seem to be more vulnerable to cold temperatures. In Spain, as in most of the European countries, women have more sickness absence than men, which has been related to the gendered division of paid work and family responsibilities and the related women's double presence (33). Moreover, after the birth of their first child, within-couple gender gap in sickness increases comparing to pre-child gap, a difference that remains up until 15 years after the birth of the first child (34). Furthermore, women are exposed to a higher level of precarious employment and working conditions, which have been shown to be a predictor of sickness absence episodes (35). But none of this explanation wouldn't justify the fact that women are more vulnerable only to cold temperatures. Physiological sex differences (such as body mass or body fat) may also contribute to the sex-related differences in susceptibility to temperature (36).

Several previous studies conducted in different countries have generally shown that socioeconomic status is associated with the risk of sickness absence, people in a lower occupational class have more SA than those in a higher occupational class (37, 38). Unexpectedly, we found an association between cold temperatures and risk of sickness absence only among non-manual workers. This counterintuitive result is in line with the findings of other research on temperature and occupational injuries (12), and with some research analyzing sickness absence by occupational class which find that non-manuals had more sickness absence than manual workers among women (39) and younger employees (40) in particular. A possible explanation is that non-manual workers perform their work mostly indoors, thus being more exposed to interpersonal transmission of respiratory virus, also they could have an easier access to health care services. Nevertheless, this hypothesis does not explain the absence of association of among manual workers. More research is needed in order to understand this complex reality.

### Limitations and strengths

This study has some limitations. First, we are probably underestimating the association between exposure to extreme temperatures and temporary disability, due to the economic sector composition of the workers in our sample, i.e., mainly service sector workers, and few workers in agriculture and construction, who are likely to be most exposed to high risks of adverse health outcomes from heat. Second, the lack of information on whether workers perform their work indoors or outdoors, and about the specific occupation of workers and their working conditions, which could shed light to the effect of ambient non-optimal temperatures depending working conditions of each occupation; even though we have economic sector and occupational category as proxies. Third, we do not have information on living conditions, which also could be confounding the effect of temperature and sickness absence on working population. Finally, we did not have information about comorbidities or previous health status. Among the strengths of this study, this is the first one assessing the impact of ambient temperatures on workers' health through a holistic measure such as sickness absence. Diagnoses of sickness absence are not self-reported, we have official medical diagnoses made by primary care doctors. Finally, we used administrative secondary data from social security and sickness absence management institutions at cost zero for use in this research.

### Conclusions

As far as we know, there is no cross-national survey in Europe on working conditions that incorporates items on the basis of a theoretical approach to the impact of climate crisis to occupational health. So, there is a lack of information and indicators of how the climate crisis threats workers' health. Both occupational health surveillance and research are needed to identify climate-related occupational effects in order to develop preventive strategies and workplaces adaptations for vulnerable groups within working population. However, these preliminaries results can provide guidance on the definition and implementation of policies to prevent potential risks of temperature to workers health, specifically cold temperatures, which have not been usually taken into consideration.

## Data availability statement

The data analyzed in this study is subject to the following licenses/restrictions: The data that support the findings of this study are available from the Spanish National Social Security Institute and the Catalonian Institute for Medical Evaluations but restrictions apply to the availability of these data, which were used under license for the current study, and so are not publicly available. Data are however available from the authors upon reasonable request and with permission of the Spanish National Social Security Institute and the Catalonian Institute for Medical Evaluations. Requests to access these datasets should be directed to AA-G, amaya.ayala@upf.edu.

## Ethics statement

Ethical review and approval was not required for the study on human participants in accordance with the local legislation and institutional requirements. Written informed consent for participation was not required for this study in accordance with the national legislation and the institutional requirements.

## Author contributions

XB, AA-G, MU, and FB conceived the article. XB analyzed the data. MU, AA-G, and XB interpreted the data and results. MU and AA-G drafted the work. XB and FB substantively revised it. All authors contributed to the article and approved the submitted version.
