# Supplementary material for: Extreme temperatures and sickness absence in the Mediterranean province of Barcelona: An occupational health issue
Source: Front Public Health. 2023 Feb 20;11:1129027. doi: 10.3389/fpubh.2023.1129027 (PMC9986628; doi:10.3389/fpubh.2023.1129027)

Supplementary Material

Extreme temperatures and sickness absence in the Mediterranean province of Barcelona: an occupational health issue

Mireia Utzet[1,2,3], Amaya Ayala-garcia[2,1,3*], Fernando G. Benavides[1,2,3], Xavier Basagaña[4,5]

*** Correspondence:** Amaya Ayala-Garcia: amaya.ayala@upf.edu

Figure S1. Relationship between average temperature (ºC) and risk of sickness absence due to respiratory system diseases in Barcelona for the period 2012-2015 with 95% confidence intervals. a) Cumulative Relative Risks (RR) for lags 0-7. The dashed lines indicate the 5th and 95th percentiles of average temperature. The dotted line indicates the temperature of minimum risk. b) Association of cold temperatures (RR for 5th percentile of temperature compared to temperature of minimum risk) by lag. c) Association of hot temperatures (RR for 95th percentile of temperature compared to temperature of minimum mortality) by lag.

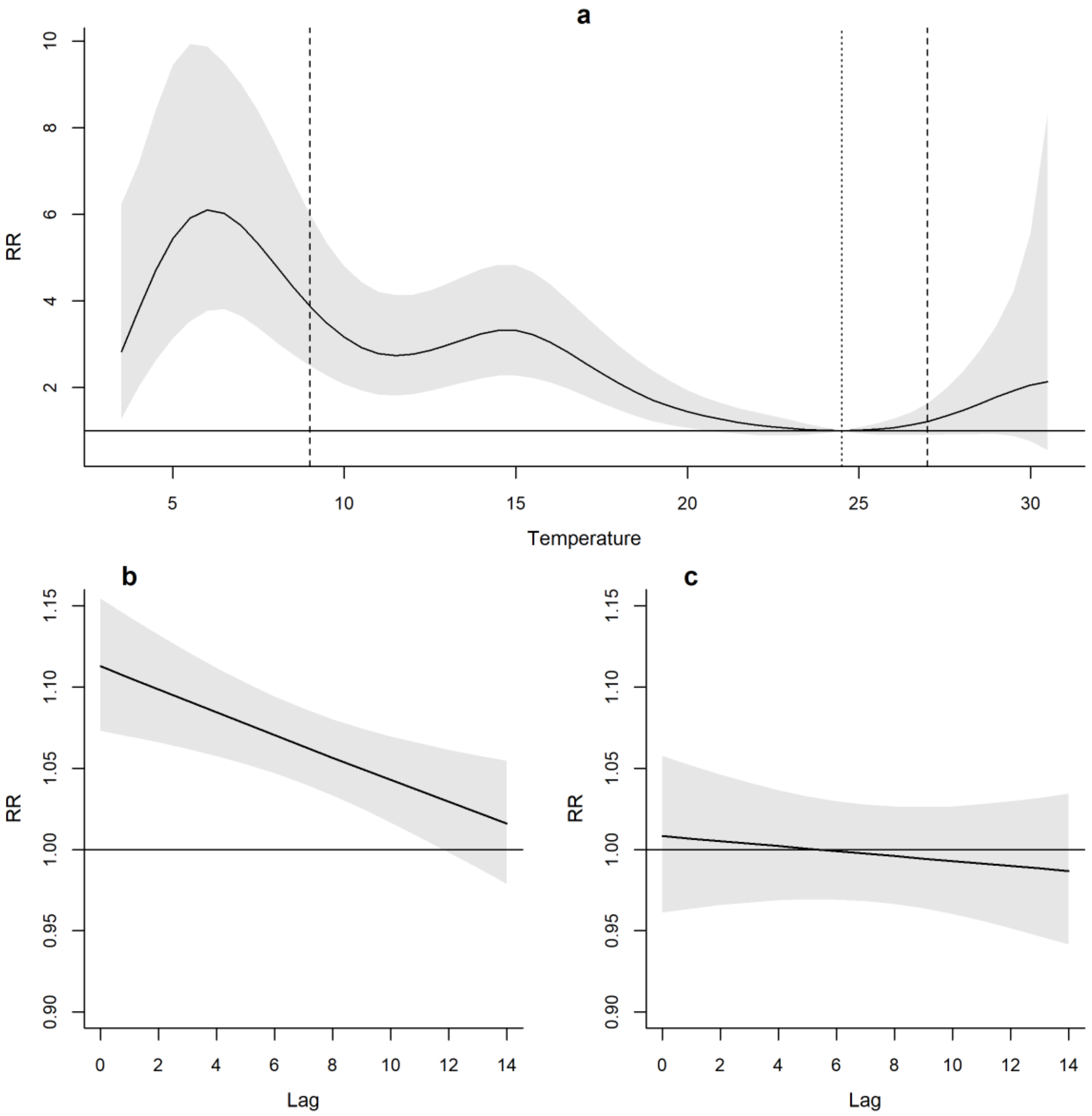
a
RR
10
8
6
4
2
5
10
15
20
25
30
Temperature
b
RR
1.15
1.10
1.05
1.00
0.95
0.90
0
2
4
6
8
10
12
14
Lag
c
RR
1.15
1.10
1.05
1.00
0.95
0.90
0
2
4
6
8
10
12
14
Lag

Figure S2. Relationship between average temperature (ºC) and risk of sickness absence due to infectious diseases in Barcelona for the period 2012-2015 with 95% confidence intervals. a) Cumulative Relative Risks (RR) for lags 0-7. The dashed lines indicate the 5th and 95th percentiles of average temperature. The dotted line indicates the temperature of minimum risk. b) Association of cold temperatures (RR for 5th percentile of temperature compared to temperature of minimum risk) by lag. c) Association of hot temperatures (RR for 95th percentile of temperature compared to temperature of minimum mortality) by lag.

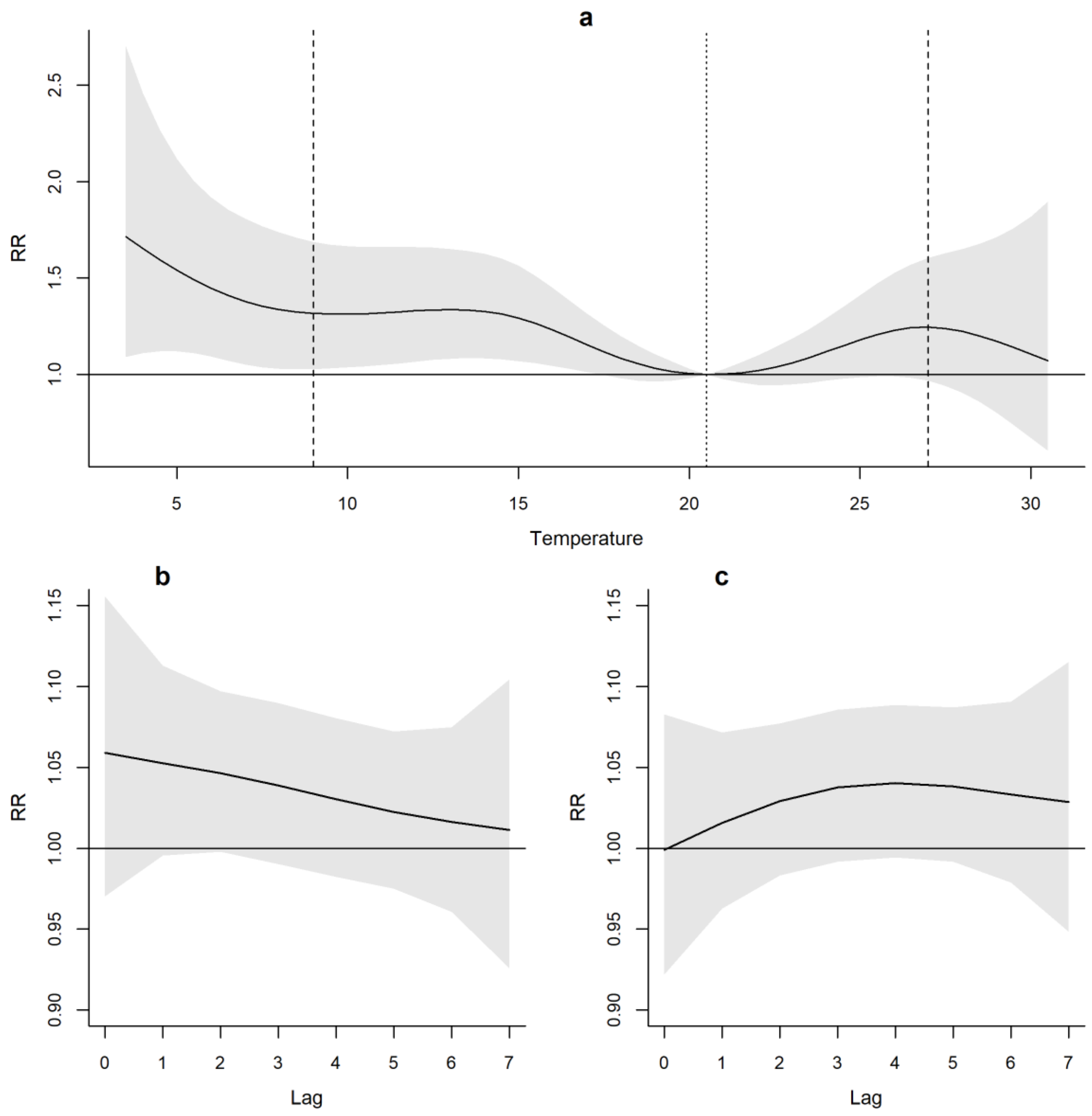

Supplement: Supplementary file 1 [file Data_Sheet_1.PDF]
